# The association between glucometabolic disturbances, traditional cardiovascular risk factors and self-rated health by age and gender: A cross-sectional analysis within the Malmö Preventive Project

**DOI:** 10.1186/1475-2840-10-118

**Published:** 2011-12-28

**Authors:** Margret Leosdottir, Ronnie Willenheimer, Margaretha Persson, Peter M Nilsson

**Affiliations:** 1 Department of Cardiology, Skåne University Hospital, Malmö, Sweden; 2 Heart Health Group and Lund University, Malmö, Sweden; 3 Department of Clinical Sciences, Lund University, Skåne University Hospital Malmö, Sweden

**Keywords:** Age, cardiovascular disease, diabetes mellitus, gender, glucose, self-rated health

## Abstract

**Background:**

The increased risk of cardiovascular disease (CVD) in diabetic compared to non-diabetic subjects seems to decrease with age. Whether this age-related reduction applies to CVD risk factors, and whether it is limited to established diabetes mellitus (DM) or also applies to pre-diabetic conditions are not well known.

**Methods:**

Using a cross-sectional design we compared the strength of the correlation between glucometabolic disturbances (by grouping), CVD risk factor burden and self-rated health, in two age groups: middle-aged (57-69 years) and older (70-86 years) subjects, (63% men), participating in the Malmö Preventive Project Re-examination Study (n = 18,238). Simple (unadjusted) logistic regression analysis was applied to estimate between-group differences and trends. Interaction analysis was applied to estimate differences between age groups.

**Results:**

CVD risk factor burden and the proportion of subjects reporting poor self-rated health increased with increasing glucometabolic disturbance for men and women in both age groups (p-trend < 0.0001 for all). The slope of the trend curve with increasing CVD risk factor burden was significantly steeper for older women than for older men (p-interaction = 0.002). The slope of the trend curve for poor self-rated health was significantly steeper for middle-aged than for older men (p-interaction = 0.005), while no difference was observed between the age groups among women (p-interaction = 0.97).

**Conclusions:**

We found no reduction in risk factor accumulation with increasing glucometabolic disturbance between middle-aged and older subjects. Our results indicate life-long CVD risk factor clustering with increased glucometabolic disturbance, and suggest that previously observed age-related reduction in excess CVD risk for subjects with DM might be due to a survival bias. However, our observations indicate more pronounced risk factor clustering and worse self-rated health with increased glucometabolic disturbance in older women than in older men.

## Background

Diabetes mellitus (DM) is a well-established risk factor for cardiovascular disease (CVD). The Framingham Study was the first to describe a 2-3 fold increase in the risk of CVD in diabetic compared to non-diabetic subjects, with women bearing a disproportionate burden of the risk [1]. Fasting glucose level below the diabetes threshold has also been shown to be a risk factor for CVD [2]. Prevention and treatment of glucometabolic disturbances is thus important, and has been recommended as a means of reducing the future risk of CVD complications [3].

The incidences of glucometabolic disturbances and CVD increase with age. However, it has been shown that the increased incidence of CVD in diabetic compared to non-diabetic subjects seems to decrease with increasing age [4]. Whether this age-related reduction is limited to those with established DM or also applies to subjects with pre-diabetic conditions is less well known. Also, studies focusing on gender differences are lacking. It is important to explore these aspects as they could affect treatment strategies. In the current analysis we compared the strength of the correlation between glucometabolic status - defined by fasting plasma glucose (FPG) value and a clinical diagnosis of impaired glucose metabolism - and CVD risk factors, in two age groups (middle-aged and older) of men and women, participating in the Malmö Preventive Project Re-examination Study. We hypothesized that a corresponding age-related reduction would be observed across the spectrum of glucometabolic disorders. As a perceived measure of health we also examined whether the strength of the correlation between glucometabolic status and self-rated health (SRH) differed between the age groups.

## Methods

The Malmö Preventive Project was a population-based cohort study conducted between 1974 and 1992. The project was a preventive case-finding program with the aim of screening for CVD risk factors, alcohol abuse and breast cancer in the population [5]. Birth cohorts of inhabitants of Malmö, the third largest city in Sweden, were invited to participate (men born in the years 1921, 1926-42, 1944, 1946 and 1948-9; women born in 1926, 1928, 1930-6, 1938, 1941-2 and 1949). Approximately 33,000 individuals participated (71% participation rate) of whom two-thirds were men. The screening process has been described in detail elsewhere [5,6].

The Malmö Preventive Project Re-examination Study was conducted at Skåne University Hospital in Malmö during the period 2002-2006. The target population consisted of the approximately 25,000 individuals in the original Malmö Preventive Project cohort, still alive and living in the Malmö area. In total, 18,238 subjects participated (63% men) giving a participation rate of approximately 72%. At the first visit the subjects were given verbal information on the study, and blood samples were drawn (after overnight fasting). Laboratory tests included FPG, serum (s-) total cholesterol, s-triglycerides and s-high-density lipoprotein (Beckman Coulter LX20, Beckman Coulter Inc., Brea, USA). Low-density lipoprotein cholesterol was calculated using the Friedewald formula [7]. Whole blood was drawn and stored in a biobank for later genetic analyses [8]. At the second visit (a week later) blood pressure and pulse rate were measured twice in the supine position after 5 minutes' rest, by trained nurses. Height (m) and weight (kg) were measured in light indoor clothing without shoes. Waist and hip circumference (cm) were measured. The participants then answered a questionnaire on lifestyle, self-assessment of health, limited medical history, and medication. If the FPG measured at the first visit was elevated (≥ 7.0 mmol/L), new blood samples were drawn on the second visit. In a sub-sample of participants (n = 1792) echocardiography and ECG recording were performed, and levels of s-Nt proBNP, s-cystatin C and HbA_1c _were measured at a separate visit [9].

Data quality was high, with missing values ranging from 0.1% (laboratory measures) to 0.5% (anthropometric measures, blood pressure and pulse rate) to 1.5% (questionnaire data). All participants received information on the results of their laboratory tests and blood pressure measurements, and were offered an appointment with a physician in cases of new-onset type-2 DM (T2DM), dyslipidemia or hypertension, if they did not have a personal family physician.

The Ethics Committee of Lund University, Sweden, approved the Malmö Preventive Project Re-examination Study (No. LU 244-02). The study complied with the Declaration of Helsinki. All participants signed an informed consent form before entering the study.

### Description of variables

In the current study, the subjects were divided into six groups according to glucometabolic status. In the first three groups the FPG was within the normal range (≤ 5.0 mmol/L; 5.1-5.5 mmol/L; 5.6-6.0 mmol/L). The 4^th ^group constituted subjects with impaired fasting glucose (IFG) according to the World Health Organization (WHO) criteria (FPG 6.1-6.9 mmol/L) [10], the 5^th ^subjects with new-onset T2DM, and the 6^th ^group patients with established DM (T1DM or T2DM). DM was classified as established if self-reported in the questionnaire (T1DM or T2DM) and/or if the subject was taking prescription drugs for DM. New-onset T2DM was defined by two separate FPG values ≥ 7.0 mmol/L [10]. Additionally, a single measurement ≥ 11.1 mmol/L was classified as new-onset DM. One measurement of FPG 7.0-11.0 mmol/L (the second measurement being ≤ 6.9 mmol/L) was included in the 4^th ^group. This was done to maximize the benefit of having two separate measurements and thus minimize the risk of mis-classification.

Five CVD risk factors were chosen for this study: the presence of uncontrolled hypertension, dyslipidemia, central obesity, current smoking and lack of physical activity. Uncontrolled hypertension was defined as systolic blood pressure ≥ 140 mmHg and/or diastolic blood pressure ≥ 90 mmHg, irrespective of hypertension treatment. Dyslipidemia was defined as at least one of the following: total cholesterol ≥ 5.0 mmol/L, low-density lipoprotein ≥ 3.0, triglycerides ≥ 1.7 mmol/L and/or high-density lipoprotein < 1.0 mmol/L for men and < 1.3 mmol/L for women. Central obesity was defined as a waist circumference of ≥ 102 cm for men and ≥ 88 cm for women. These limits were based on the European Society of Cardiology guidelines for CVD prevention (blood pressure, total cholesterol, low-density lipoprotein) and classification of the metabolic syndrome (triglycerides, high-density lipoprotein, waist circumference) [11,12]. Current smoking and physical activity were self-reported in the questionnaire. Those reporting no leisure time physical activity were classified as being physically inactive. SRH was measured by questionnaire using the standardized question: "Would you say that in general your health is... excellent, very good, good, fair or poor?" [13]. Subjects were classified as having low SRH when answering fair or poor.

### Statistical analysis

All calculations were stratified by gender and age: middle-aged (57-69 years) and older (70-86 years). The proportions (%) of subjects having at least three of the five selected risk factors and low SRH were calculated for each of the six glucometabolic status groups described above. Differences in proportions between age groups within each glucometabolic status group were calculated by using simple (unadjusted) logistic regression, generating odds ratios (OR) and 95% confidence intervals (95% CI). To assess the strength of the correlation between increasing glucometabolic disturbance and increased risk factor burden and worse SRH, logistic regression analysis was used to calculate beta (β)-coefficients for trends from the 1^st ^to the 6^th ^group for SRH and from the 1^st ^to the 5^th ^group for the risk factors. The 6^th ^group (established DM) was excluded in the latter case as non-linearity could be expected, as the result of better diagnosis and treatment of risk factors for subjects with established DM. Ratios between the β-coefficients obtained from the trend analyses were then calculated, and interaction analysis was performed to estimate differences between the age groups, by entering variable A *variable B into the regression analysis. A traditional double-sided significance level of p < 0.05 was used. Baseline characteristics are presented as means ± standard deviations and percentages. The SPSS 19.0 computer package was used for statistical analysis (SPSS Inc, IL, USA).

## Results

The total Malmö Preventive Project Re-examination Study cohort (n = 18,238) was used in this study. Twelve non-diabetic subjects lacked FPG values and could thus not be categorized. Baseline characteristics for men and women are given in Table 1. For simplification, data from the first three glucometabolic groups were combined in the table as these subjects all had normal fasting glucose (NFG) levels.

**Table 1:** Baseline characteristics (presented as means ± SD and percentages (%)) of the participants in the Malmö Preventive Project Re-examination Study.

	Middle-aged (57-69 years)				Older (70-86 years)			
Men (N = 11,546)	NFG	IFG	New-onset DM	Established DM	NFG	IFG	New-onset DM	Established DM
Number (%)	4977 (70)	1299 (18)	158 (2)	721 (10)	3002 (68)	777 (18)	85 (2)	527 (12)
Mean age (years)	64 ± 4	64 ± 4	63 ± 3	65 ± 3	75 ± 3	75 ± 3	74 ± 3	75 ± 3
SBP (mmHg)	143 ± 19	150 ± 19	157 ± 20	148 ± 19	145 ± 19	152 ± 20	151 ± 23	148 ± 20
DBP (mmHg)	84 ± 11	88 ± 10	90 ± 10	84 ± 10	83 ± 11	85 ± 10	86 ± 11	81 ± 11
Waist circumference (cm)	97 ± 10	102 ± 11	108 ± 12	105 ± 12	97 ± 10	102 ± 10	104 ± 9	103 ± 11
Total cholesterol (mmol/L)	5.5 ± 1.0	5.6 ± 1.1	5.7 ± 1.1	4.8 ± 1.1	5.4 ± 1.0	5.3 ± 1.0	5.3 ± 1.0	4.7 ± 1.0
LDL (mmol/L)	3.7 ± 0.9	3.7 ± 1.0	3.7 ± 1.0	3.0 ± 0.9	3.5 ± 0.9	3.4 ± 0.9	3.3 ± 0.9	2.9 ± 0.9
HDL (mmol/L)	1.3 ± 0.4	1.2 ± 0.3	1.1 ± 0.3	1.1 ± 0.3	1.4 ± 0.4	1.3 ± 0.4	1.3 ± 0.3	1.2 ± 0.3
Triglycerides (mmol/L)	1.2 ± 0.8	1.5 ± 1.1	2.1 ± 1.6	1.7 ± 1.3	1.1 ± 0.6	1.4 ± 0.8	1.7 ± 0.9	1.5 ± 0.9
Current smoker (%)	21.1%	21.1%	25.3%	17.3%	13.5%	13.3%	15.3%	10.4%
Physically inactive (%)	8.5%	10.9%	21.7%	16.9%	9.4%	11.9%	15.5%	18.8%
Using medication for CVD or HTN (%)	35%	44%	45%	75%	53%	62%	65%	84%

**Women (N = 6680)**								
Number (%)	2979 (84)	331 (9)	28 (1)	202 (6)	2517 (80)	333 (11)	45 (1)	245 (8)
Mean age (years)	66 ± 4	66 ± 3	67 ± 2	67 ± 3	73 ± 3	74 ± 3	74 ± 3	74 ± 3
SBP (mmHg)	140 ± 20	146 ± 20	147 ± 23	145 ± 21	146 ± 21	151 ± 22	157 ± 24	146 ± 20
DBP (mmHg)	82 ± 10	85 ± 11	86 ± 9	82 ± 10	82 ± 10	85 ± 11	87 ± 11	81 ± 10
Waist circumference (cm)	86 ± 11	93 ± 12	100 ± 13	97 ± 14	86 ± 11	93 ± 12	97 ± 11	96 ± 13
Total cholesterol (mmol/L)	6.0 ± 1.0	6.0 ± 1.0	6.2 ± 1.4	5.3 ± 1.1	6.0 ± 1.1	5.9 ± 1.1	6.2 ± 1.2	5.1 ± 1.0
LDL (mmol/L)	3.9 ± 1.0	3.9 ± 0.9	4.1 ± 1.3	3.2 ± 0.9	3.8 ± 1.0	3.7 ± 1.0	4.0 ± 1.0	3.0 ± 0.9
HDL (mmol/L)	1.6 ± 0.4	1.4 ± 0.4	1.2 ± 0.3	1.4 ± 0.4	1.7 ± 0.4	1.5 ± 0.5	1.3 ± 0.4	1.4 ± 0.4
Triglycerides (mmol/L)	1.1 ± 0.6	1.5 ± 0.7	2.1 ± 0.9	1.6 ± 1.6	1.2 ± 0.5	1.4 ± 0.7	1.8 ± 0.9	1.6 ± 0.9
Current smoker (%)	18.5%	21.1%	21.4%	14.4%	12.2%	15.3%	11.1%	11.4%
Physically inactive (%)	8.0%	11.3%	21.4%	19.3%	11.5%	20.4%	27.3%	20.4%
Using medication for CVD or HTN (%)	32%	51%	54%	69%	48%	59%	49%	82%

NFG, normal fasting glucose; IFG, impaired fasting glucose; DM, diabetes mellitus; SBP, systolic blood pressure; DBP, diastolic blood pressure; HDL, high-density lipoprotein, LDL, low-density lipoprotein; HDL, high-density lipoprotein; CVD, cardiovascular disease; HTN, hypertension.

### Risk factor burden

**Figure 1:**
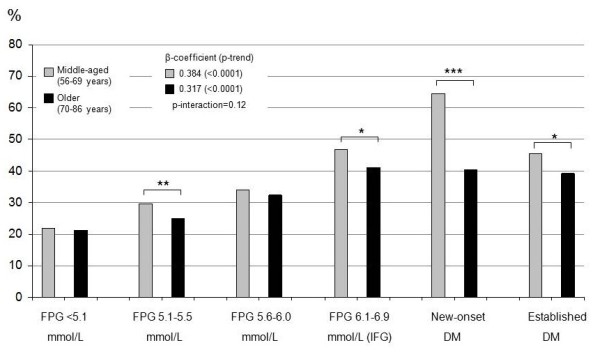
**Proportion of men with three or more risk factors**. The figure shows the proportion of *men *with three or more of the following risk factors: uncontrolled hypertension, dyslipidemia, central obesity, current smoker or being physically inactive; in each of the six glucometabolic status groups defined in the text. β-coefficients and p-values for trends from the 1^st ^to the 5^th ^groups are also shown. Brackets denote differences between age groups within each glucometabolic status group: * p < 0.05; ** p < 0.01; *** p < 0.001.

The proportions (%) of subjects within each glucometabolic status group and age group having at least three of the five selected risk factors are shown in Figures 1 (men) and 2 (women). For the whole cohort and each glucometabolic group, women in general exhibited more risk factors than men, also after adjustment for age. There was a highly significant trend among both men and women for increasing risk factor burden from the 1^st ^through the 5^th ^glucometabolic group in both age groups (p-trend < 0.0001). The ratio between the β-coefficients from the trend tests (reflecting the slope of the curve) for middle-aged vs. older men was 1.21 (p-interaction = 0.12) and for women 0.97 (p-interaction = 0.78). Although the difference in trends between age groups was not significant among men, older subjects with FPG = 5.1-5.5 mmol/L (OR 0.78 (95% CI 0.67-0.92) p = 0.002), IFG (0.80 (0.67-0.96), p = 0.02), new-onset T2DM (0.37 (0.22-0.65), p = 0.0004) and established DM (0.78 (0.62-0.98), p = 0.03) had significantly fewer risk factors than the middle-aged subjects in the respective groups. No such differences between age groups were seen among women. The ratio between β-coefficients for middle-aged women vs. middle-aged men was 1.19 (p-interaction = 0.11) while for older women vs. older men it was 1.49 (p-interaction = 0.002).

**Figure 2:**
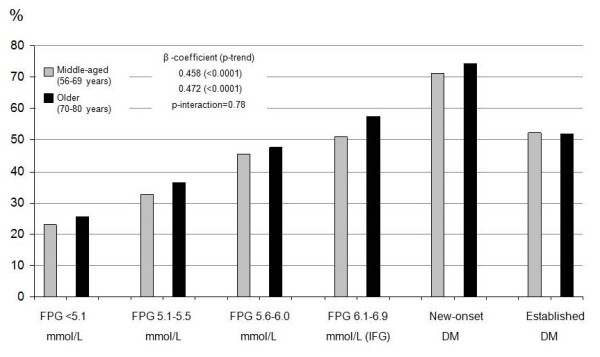
**Proportion of women with three or more risk factors**. The figure shows the proportion of *women *with three or more of the following risk factors: uncontrolled hypertension, dyslipidemia, central obesity, current smoker or being physically inactive; in each of the six glucometabolic status groups defined in the text. β-coefficients and p-values for trends from the 1^st ^to the 5^th ^groups are also shown.

### Self-rated health

**Figure 3:**
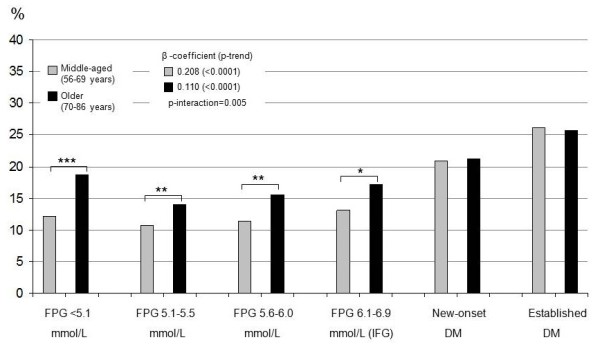
**Proportion of men reporting low self-rated health**. The figure shows the proportion of *men *reporting low SRH for each of the six glucometabolic status groups defined in the text. β-coefficients and p-values for trends from the 1^st ^to the 6^th ^groups are also shown. Brackets denote differences between age groups within each glucometabolic status group. * p < 0.05; ** p < 0.01; *** p < 0.001.

The proportions (%) of subjects within each glucose group and age group reporting low SRH are presented in Figures 3 (men) and 4 (women). On average, women and older subjects of both sexes more often reported low SRH. The middle-aged men with NFG (first three groups) and IFG (4^th ^group) reported significantly better SRH than older men in these groups, while there was no difference in SRH between middle-aged and older men with new-onset and established DM. Consequently, the trend from the 1^st ^to the 6^th ^groups for the middle-aged men was stronger (β-coefficient = 0.21, p-trend < 0.0001) than for the older men (β-coefficient = 0.11, p-trend < 0.0001), giving a ratio of 1.89 (p-interaction = 0.005). For women, the β-coefficient for middle-aged women was 0.17 (p-trend < 0.0001) compared to 0.14 (p-trend < 0.0001) for older women; the ratio being 1.22 (p-interaction = 0.47). The differences in β-coefficients between men and women in each age group were not statistically significant (middle-aged: β-coefficient ratio 0.82, p-interaction = 0.33; older: β-coefficient ratio 1.26, p-interaction = 0.47).

**Figure 4:**
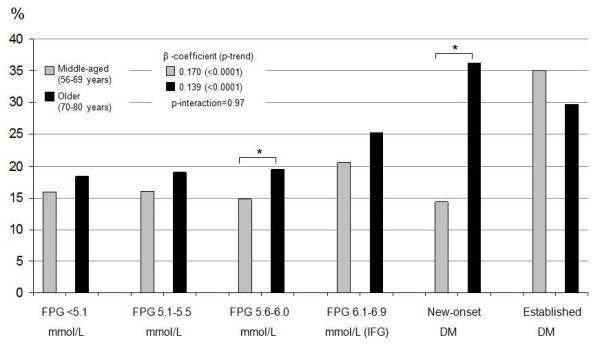
**Proportion of women reporting low self-rated health**. The figure shows the proportion of *women *reporting low SRH for each of the six glucometabolic status groups defined in the text. β-coefficients and p-values for trends from the 1^st ^to the 6^th ^groups are also shown. Brackets denote differences between age groups within each glucometabolic status group. * p < 0.05.

## Discussion

In this population-based cohort study of middle-aged and older subjects, we observed that CVD risk factor burden increased linearly with increasing glucometabolic disturbance among men and women in both age groups. The slope of the trend curve was not significantly steeper for middle-aged than older subjects of either sex, indicating that there was no significant attenuation of the relation between increasing glucometabolic disturbance and CVD risk factor burden with age. However, older men with IFG, new-onset DM and established DM had significantly fewer risk factors than middle-aged men in the corresponding glucometabolic status groups; no such difference was observed among women. Consequently, the slope of the trend curve was significantly steeper for older women than older men, indicating a higher risk factor accumulation with increasing glucometabolic disturbance among the older women. We observed that older subjects of both genders reported worse SRH than middle-aged subjects. This difference decreased with increasing glucometabolic disturbance among men, but not among women.

Although the excess CVD risk due to DM in women compared to men seems to have declined since first described by the Framingham investigators in the 1970s, recent studies still show that women bear a disproportionate part of the DM-related excess risk [14,15]. There have been speculations as to the reasons for this difference. In the INTERHEART study, DM contributed more to the population-attributable risk of myocardial infarction in women than men [16]. Disparities in diagnosis and treatment of CVD, as well as gender-related differences in response to treatment have been another suggested explanation [17]. It has also been suggested that there is a higher CVD risk factor burden in diabetic women than in diabetic men [18]. Our study confirms this, but women in all glucometabolic status groups in both age groups exhibited more CVD risk factors than men. The same trend was observed for SRH, in itself a strong predictor of all-cause and disease-specific mortality, also among subjects with DM [19-21].

We have not been able to find any studies reporting age- and gender-stratified CVD risk ratios or risk factor burdens across the spectrum of glucometabolic disturbances. Several studies on CVD risk ratios between diabetic and non-diabetic subjects have, however, been reported, some of which have presented gender-specific, age-stratified results. A Norwegian study on coronary heart disease mortality showed a decline in risk ratio from the youngest (< 60 years) to the oldest (> 80 years) age groups, equally so for men and women [4]. In the Nurses Health Study, however, only a minimal difference in coronary heart disease mortality risk ratios was found between women with and without DM in the age ranges 55-64 vs. > 65 years [22]. Likewise, in the NHANES survey from 1999, coronary heart disease mortality risk ratios between diabetic and non-diabetic men halved between the age groups 55-64 years and 65-74 years, while they remained unchanged for women [23]. Although we analyzed surrogate enpoints cross-sectionally in the present study, and it is therefore not fully comparable to these studies, our results concur with the two latter studies regarding subjects with DM only. As such, the older men with new-onset and established DM had significantly fewer risk factors than middle-aged men in the respective groups while no such difference was seen among the women. This lack of age-related reduction in risk factor clustering and subsequently hypothetical risk of CVD morbidity/mortality among diabetic women may make a contribution to the observed difference in excess CVD risk for diabetic women compared to men.

In our study, the risk factor burden increased significantly across the glucometabolic status groups for men and women in both age groups. Although there were significant differences between middle-aged and older men with IFG, new-onset and established DM as discussed above, the difference in trends for risk factor accumulation across glucometabolic status groups between the two age groups was not significant for either sex. This discrepancy might reflect a survival bias, in that subjects with glucometabolic disturbances experience a life-long tendency for risk factor clustering while those with the most serious metabolic disturbances succumb to CVD events at an earlier age. However, it should be noted that the trend was significantly weaker among older men than older women, who showed an accumulation of risk factors with increasing glucometabolic disturbance to the same degree as both middle-aged women and men. These results support the fact that not only women with DM but also prediabetic women, irrespective of age, need special attention when screening for and treating concomitant CVD risk factors.

The major strength of this study is that it examines the association between CVD risk factor profile and degree of glucometabolic disturbance through the range from NFG to IFG and DM, in a large population-based cohort, allowing comparison between different groups and the assessment of trends and interactions. The participation rate in the study was high (72%) and missing values were few, increasing the validity of the results.

There are some limitations in this study. Firstly, as with all population-based cohort studies, there is risk of a "healthy cohort" effect, meaning that subjects with co-morbidities, disabilities or poor quality of life might either have died or been too weak to participate in the study. As these subjects would be more likely to be older and classified in the groups of subjects with higher glucometabolic disturbances, such a bias would lead to underestimation of CVD risk factor burden and poor SRH in those groups. A study on attendees versus non-attendees was performed in the original Malmö Preventive Project, showing that total and cause-specific mortality was higher in non-participants than in those participating in the study [6]. It can be assumed that similar findings would be found in the Malmö Preventive Project Re-examination Study. Secondly, it cannot be ruled out that some non-fasting values were used for the classification of glucometabolic disturbances. Also, no oral glucose tolerance test was performed. This might have led to mis-classification of glucometabolic disturbances and underestimation of glucometabolic-related risk, especially for women as impaired glucose tolerance and DM defined by an oral glucose tolerance test is more common among women than men [3]. Thirdly, the duration of diabetes was not considered in this study. Other studies have shown that subjects with early-onset DM run a higher risk of CVD than those who develop the disease in middle age [22,24]. Gender differences in the three above-mentioned limitations, which would have led to a biased result, cannot be ruled out. Regarding risk factors for CVD rather than CVD morbidity and mortality limits the conclusions that can be drawn concerning the clinical relevance of the results. Furthermore, no conclusions about causality can be drawn from a cross-sectional study.

In conclusion, the previously observed age-related reduction in elevated risk of CVD in diabetic compared to non-diabetic subjects was not observed for risk factor accumulation with increasing glucometabolic disturbance. Our results indicate life-long risk factor clustering with increasing glucometabolic disturbance, while an age-related CVD risk attenuation with respect to DM might be due to a survival bias. However, our observations suggest a more pronounced risk factor clustering and a more negative effect on SRH with increasing glucometabolic disturbance in older women than in older men. These results may contribute to the previously observed higher risk of CVD in women with glucometabolic disturbances than in men. The results also indicate the need to pay special attention to screening for and treating concomitant CVD risk factors among women with glucometabolic disturbances, irrespective of age.

## List of Abbreviations used

CVD: cardiovascular disease; DM: diabetes mellitus; FPG: fasting plasma glucose; IFG: impaired fasting glucose; SRH: self-rated health; T1DM: Type-1 diabetes mellitus; T2DM: Type-2 diabetes mellitus.

## Competing interests

The authors declare that they have no competing interests.

## Authors' contributions

ML designed the study in collaboration with PN and RW. ML carried out the statistical analysis and drafted the manuscript. MP was in charge of data collection in the Malmö Preventive Project Re-examination Study. RW, MP and PN critically revised the manuscript for intellectual content. All authors read and approved the final manuscript.
